# Complete Genetic Analysis of Plasmids Carrying Multiple Resistance, Virulence, and Phage-Like Genes in Foodborne Escherichia coli Isolate

**DOI:** 10.1128/spectrum.02820-22

**Published:** 2023-03-21

**Authors:** Xiaobo Liu, Ruichao Li, Edward Wai-Chi Chan, Sheng Chen

**Affiliations:** a National Engineering Laboratory for Deep Process of Rice and By-Products, College of Food Science and Engineering, Central South University of Forestry and Technology, Changsha, Hunan, China; b Institute of Comparative Medicine, College of Veterinary Medicine, Yangzhou University, Yangzhou, Jiangsu Province, People’s Republic of China; c The State Key Lab of Chemical Biology and Drug Discovery, Department of Applied Biology and Chemical Technology, The Hong Kong Polytechnic University, Kowloon, Hong Kong SAR; d Department of Infectious Diseases and Public Health, Jockey Club College of Veterinary Medicine and Life Sciences, City University of Hong Kong, Kowloon, Hong Kong SAR; e City University of Hong Kong Shenzhen Research Institute, Shenzhen, China; University of Guelph College of Biological Science

**Keywords:** resistance genes, foodborne *E. coli*, phage, genetic analysis, virulence genes

## Abstract

Bacterial antimicrobial resistance, especially phenotypic resistance to multiple drugs (MDR), has posed a serious threat to public health worldwide. To clarify the mechanism of transmission of multidrug resistance encoding plasmids in *Enterobacterales*, all seven plasmids of an Escherichia coli (E. coli) strain 1108 obtained from a chicken meat sample were extracted and sequenced by Illumina Nextseq 500 and MinION platforms. Plasmids in strain 1108 possessed 16 known antimicrobial resistance genes, with p1108-NDM (~97K) being the most variable plasmid. The multidrug resistance region of p1108-NDM was punctuated by eight IS*26* insertion sequences; thus, four MDR regions were found in the backbone of this plasmid. The plasmid p1108-MCR (~65K) was found to lack the IS*Apl1* element and harbor the *bla*_CTX-M-64_-IS*Ecp1* transposition unit. Moreover, the IS*Ecp1*-*bla*_CMY-2_ transposition unit was found in plasmid p1108-CMY2 (~98K), whereas plasmid p1108-emrB (~102K) was associated with resistance to erythromycin (*emrB*) and streptomycin (*aadA22*). p1108-IncY (96K) was a phage P1-like plasmid, while p1108-IncFIB (~194K) was found to harbor a virulence region similar to ColV plasmids, and they were found to encode a conserved conjugative transfer protein but harbor no resistance genes. Finally, no mobile element and resistant genes were found in p1108-ColV (~2K). Carriage of *mcr-1*-encoding elements in carbapenemase-producing Escherichia coli will potentially render all antimicrobial treatment regimens ineffective. Enhanced surveillance and effective intervention strategies are urgently needed to control the transmission of such multidrug resistance plasmids.

**IMPORTANCE** Antimicrobial resistance (AMR) has been increasingly prevalent in agricultural and clinical fields. Understanding the genetic environment involved in AMR genes is important for preventing transmission and developing mitigation strategies. In this study, we investigated the genetic features of an E. coli strain (1108) isolated from food product and harboring 16 AMR genes, including *bla*_NDM-1_ and *mcr-1* genes encoding resistance to last line antibiotics, meropenem, and colistin. Moreover, this strain also carried virulence genes such as *iroBCDEN*, *iucABCD*, and *iutA*. Our findings confirmed that multiple conjugative plasmids that were formed through active recombination and translocation events were associated with transmission of AMR determinants. Our data warrant the continuous monitoring of emergence and further transmission of these important MDR pathogens.

## INTRODUCTION

Bacterial antimicrobial resistance, especially phenotypic resistance to multiple drugs (MDR), has posed a serious threat to human and animal health worldwide. The situation has continued to be even worse as a result of emergence of the New Delhi metallo-β-lactamase (NDM-1), which confers resistance to almost all antibiotics, including carbapenems (1). Colistin is considered one of the last-resort agents for antimicrobial treatment of serious infections caused by carbapenemase-producing *Enterobacterales* (CPE). Nevertheless, since the first discovery of horizontal transfer of the colistin resistance gene (*mcr-1*) (2), *mcr* variants (*mcr-1* to *mcr-10*) have subsequently been reported on a global scale (3, 4). More worrisome, cocarriage of *mcr* variants and carbapenemase genes (particularly *bla*_NDMs_) among *Enterobacterales*, which makes clinical treatment more difficult, heralds the advent of the era of pan-drug resistance (5, 6). Phages play an important role in mediating horizontal gene transfer between bacterial cells. Moreover, production of phage, which promotes the spread of virulence-related genes, can be induced by antibiotics (7). Until now, carriage of AMR genes in virulence plasmids has been reported in strains such as Klebsiella (8) and Salmonella (9), but there are few reports about the existence of *bla*_NDMs_ and *mcr* genes in foodborne E. coli harboring virulence and phage-like plasmids.

Here, we report the genetic characteristics of a multidrug-resistant E. coli strain recovered from chicken meat in a supermarket of Shenzhen, China in 2017. Such a strain was found to harbor as many as seven plasmids, in which 16 resistance genes were detectable, among which the plasmid carrying the *bla*_NDM-1_ gene was found to be the most variable. Findings in this work therefore provide new insights into the mechanism of transmission of MDR-encoding plasmids in *Enterobacterales*. Meanwhile, the carriage of multiple MDR plasmids in foodborne pathogens implied the risk of resistance genes transmission among food products.

## RESULTS AND DISCUSSION

Antimicrobial susceptibility tests showed that E. coli strain 1108 was resistant to most of the antimicrobials tested; however, it exhibited intermediate susceptibility to fosfomycin. Multilocus sequence typing (MLST) was performed, with results showing that the strain belonged to ST88. PCR analysis and DNA sequencing revealed that it carried the *bla*_NDM-1_, *mcr-1*, and *bla*_CTX-M-64_ genes, which presumably accounted for the corresponding drug-resistance phenotypes. The results of filter mating conjugation assays, S1-PFGE, and Southern hybridization analysis of *bla*_NDM-1_- and *mcr-1*-bearing plasmids in strain 1108 showed that the *bla*_NDM-1_- and *mcr-1*-bearing plasmids were transferable and the *bla*_NDM-1_ gene was located in a plasmid of approximately 90 kb, whereas the *mcr-1* gene was located in a plasmid with a size of ~60 kb (Fig. S1 in the supplemental material). The corresponding transconjugants harboring *bla*_NDM-1_ and *mcr-1* genes were designated MTC1108 and CTC1108, respectively. Strain MTC1108 was found to be resistant to most of the antibiotics tested but susceptible to fosfomycin, kanamycin, chloramphenicol, and nalidixic acid; however, strain CTC1108 was only resistant to colistin, cefotaxime, ampicillin, and sulfamethoxazole/trimethoprim, but susceptible to the other antibiotics.

E. coli strain 1108 was found to possess 16 known antimicrobial resistance encoding genes, matching the resistance phenotypes observed. Seven plasmids of different incompatibility types were identified and designated p1108-NDM, p1108-MCR, p1108-emrB, p1108-CMY2, p1108-IncY, p1108-IncFIB, and p1108-Col, respectively. The basic plasmid information of the seven plasmids is provided in Table 1, and p1108-NDM was the most variable plasmid among them. The complete sequences of these plasmids were subjected to BLASTN against the NCBI database to identify previously characterized plasmids for further comparative analysis.

**TABLE 1:** Genetic features of seven plasmids identified in E. coli strain 1108

Plasmid	Size (bp)	G+C (%)	Inc type	Antimicrobial resistance genes	IS elements	Accession no.
p1108-NDM	96,688	53.0	IncN	*bla*_NDM-1_, *ble*_MBL_, *sul1*, *strA*, *oqxB*, *oqxA*, *sul2*, *bla*_TEM-1_, *dfrA12*, *aadA2*, *rmtB*	IS*903B*, IS*903*, IS*Pa40*, IS*26*, IS*6100*, IS*50R*, IS*1294*, IS*Kpn26*, IS*CR1*, IS*Aba125*, IS*CR2*	MG825381
p1108-MCR	64,906	42.3	IncI2	*mcr-1*, *bla*_CTX-M-64_	IS*Ecp1*	MG825380
p1108-emrB	101,660	53.5	IncI1	*emrB*, *aadA1*	IS*26*, IS*1A*, IS*1294*	MG825377
p1108-CMY2	98,157	50.0	IncI1	*bla*_CMY-2_	IS*1294*, IS*Ecp1*, IS*Ec46*, IS*629*	MG825376
p1108-IncY	96,082	48.1	IncY	ND^*a*^	IS*1294*, IS*186B*, IS*1203*	MG825379
p1108-IncFIB	193,873	50.0	IncFIB	ND^*a*^	IS*Cro1*, IS*629*, IS*186B*, IS*2*, IS*1294*, IS*1A*, IS*640*, IS*3*, IS*Ec8*, IS*30*, IS*1A*, IS*26*	MG825378
p1108-Col	2,096	55.6	ColpVC	ND^*a*^	ND^*a*^	MH425140

a ND, not detected.

**FIG 1:**
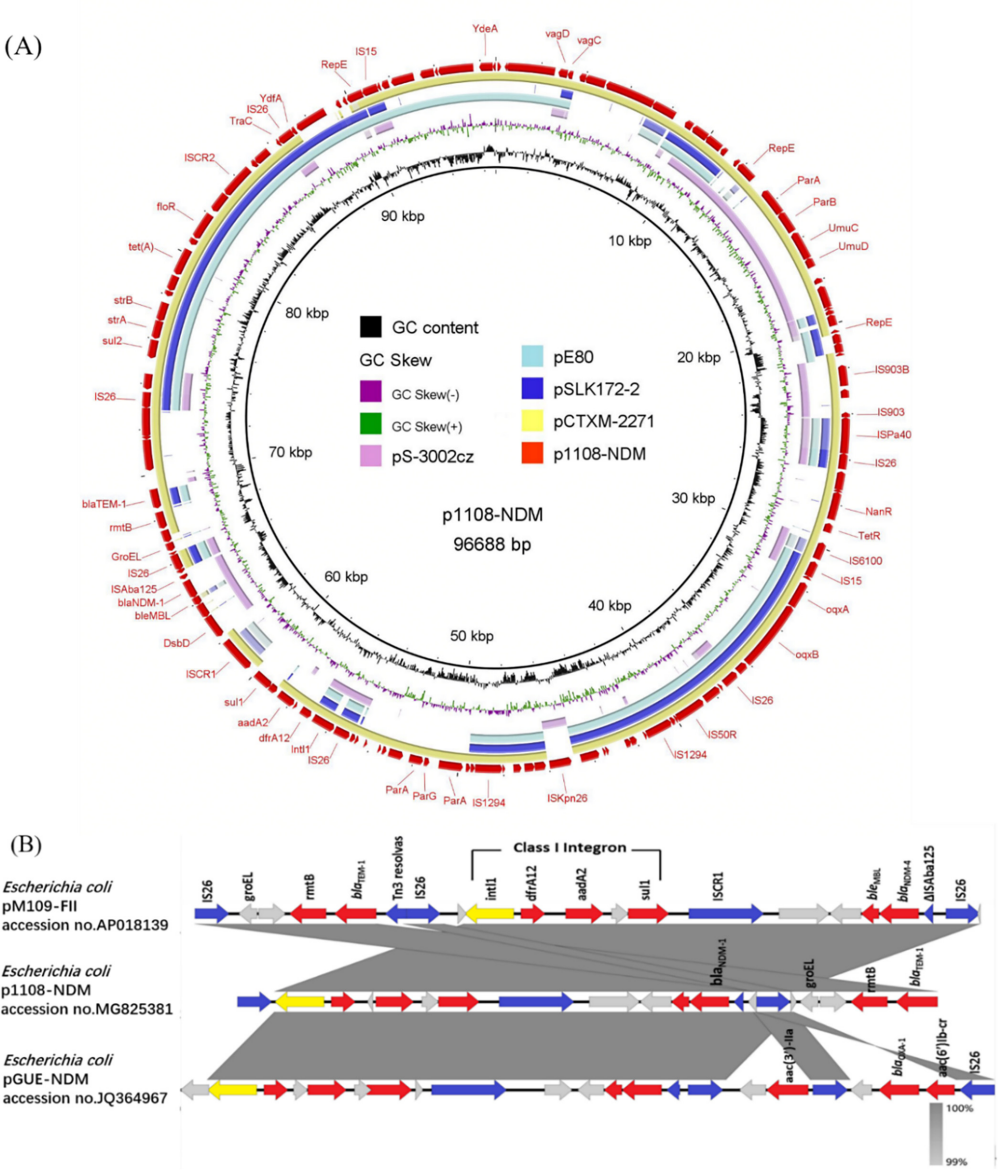
Genetic feature of *bla*_NDM-1_-bearing plasmid in E. coli strain 1108. (A) Sequence alignment of pS-3002cz (KJ958927), pE80 (KU321583), pSLK172-2 (CP017633), pCTXM-2271 (MF589339), and p1108-NDM (MG825381), with the latter being used as a reference. The outer circle with red arrows denotes annotation of the reference sequence; the gaps represent missing sequences compared to the reference plasmid. (B) Schematic representation of the structure of the *bla*_NDM-1_-surrounding sequences in plasmid p1108-NDM. Results of alignment with *bla*_NDM-1_-associated genetic structures identified in E. coli plasmid pM109-FII (AP018139) and E. coli plasmid pGUE-NDM (JQ364967) are shown. Gray shading indicates homologies between the corresponding genetic loci in each plasmid. Arrows indicate coding sequences (CDSs), with arrowheads indicating the direction of transcription: red, antibiotic resistance-encoding genes; blue, mobile elements; yellow, intl1 integrase; gray, maintenance/stability functioning genes, or hypothetical proteins.

The *bla*_NDM-1_-harboring plasmid, p1108-NDM, belongs to the IncN replication type, and additional IncR and IncX replicon genes were also identified. Several of the genes located in p1108-NDM were associated with resistance to aminoglycosides (*strA*), sulfonamides (*sul1*, *sul2*), tetracycline (*tetA*, *tetR*), penicillin (*bla*_TEM-1_), bleomycin (*ble*_MBL_), quinolone (*oqxA*, *oqxB*), phenicol (*floR*), streptomycin (*aadA2*), trimethoprim (*dfrA12*), and carbapenems (*bla*_NDM-1_) (Table 1). In addition, p1108-NDM revealed 58% query coverage and 99% nucleotide identity with an IncFII type plasmid pE80, which also harbored an IncN replicon gene (10). pE80 was carried by an E. coli strain E80 isolated from chicken meat in Hong Kong and harbored *bla*_CTX-M-55_, *oqxAB*, *fosA3*, and *bla*_TEM-1_ genes; however, the MDR region encompassing the *bla*_NDM-1_ gene was absent in it (Fig. 1A). This plasmid also exhibited sequence similarity to an IncR type plasmid pS-3002cz, which was carried by a K. pneumoniae strain in the Czech Republic (11). Interestingly, the multidrug-resistance region of p1108-NDM was punctuated by eight IS*26* insertion sequences, a key mobile element associated with transmission of antimicrobial-resistance determinants (12). Consequently, four MDR regions were found in the backbone of this plasmid, including IS*26*-*bla*_TEM-1_-*rmtB*-IS*26*, IS*26*-*oqxB*-*oqxA*-IS*26*, IS*26*-*sul2*-*strA*-*strB*-*tet(A)*-*floR*-IS*26*, and IS*26*-*intI1*-*dfrA12*-*aadA2*-*sul1*-IS*CR1*-*ble*_MBL_-*bla*_NDM-1_-ΔIS*Aba125*-IS*26*, indicating that IS*26* plays an important role in transferring the *bla*_NDM-1_ gene into the backbone. As previously stated, the *bla*_NDM_ gene was generally found in transposon Tn*125*, with two flanking IS*Aba125* mobile elements or various lengths of truncated ΔTn*125* (13). In p1108-NDM, only a small fragment of IS*Aba125* (195 bp) remains located upstream of the *bla*_NDM-1_ gene, which may suggest the genetic transmission from Acinetobacter baumannii to E. coli (14). Moreover, IS*CR1* was previously reported to be responsible for mobilization of antibiotic-resistant genes (15, 16). Here, an intact IS*CR1* element was found to be located upstream of various genetic elements containing the *trpF* phosphoribosylanthranilate isomerase gene, the bleomycin resistance gene, the *bla*_NDM-1_ gene, and the truncated transposase ΔIS*Aba125* gene. This configuration was commonly observed in plasmids harbored by NDM-1-producing Pseudomonas aeruginosa HIABP11 isolated in France (17), a strain of non-*baumannii* Acinetobacter spp. ABC7926, isolated in China (18), and a Providencia rettgeri strain pPrY2001 isolated in Canada (19); however, the backbone of these plasmids exhibited significant structural differences. Remarkably, the 14,381-bp mobile mosaic MDR region (IS*26*-*intI1-dfrA12*-*aadA2*-*sul1*-IS*CR1*-*ble*_MBL_-*bla*_NDM-1_-ΔIS*Aba125-*IS*26*) in p1108-NDM represented a class I integron designated In27 (*intI1*-*dfrA12*-*aadA2*-*sul1*), as previously reported in PKOX-P1 (KY913897) (20). Comparison analysis showed that the MDR region of p1108-NDM showed a high nucleotide identity with E. coli pM109-FII (AP018139) derived from patients’ blood specimens in Yangon, Myanmar. In *bla*_NDM-4_-bearing plasmid pM109_FII, the *bla*_TEM-1_ and *rmtB* gene cassette was bracketed by two IS*26* sequences and is located downstream of this region (21); however, it was located upstream of the *bla*_NDM-1_ gene in p1108-NDM (Fig. 1B). Moreover, pGUE-NDM (JQ364967) acquired in India also displayed a similar MDR region to p1108-NDM, with a class I integron and a module consisting of the *bla*_NDM-1_ gene, ΔIS*Aba125*, and the *ble*_MBL_ gene (22). Nonetheless, p1108-NDM lacked the large transfer region compared to pM109_FII and pGUE-NDM (Fig. 1B). Meanwhile, the *ble*_MBL_*-bla*_NDM-1_-ΔIS*Aba125* transposition unit, the *sul1* gene, and the IS*Kpn26* element were absent in pCTXM-2271, but all the other regions of p1108-NDM could be found in the backbone of pCTXM-2271, indicating that the *ble*_MBL_-*bla*_NDM-1_-ΔIS*Aba125* transposition unit was most likely captured by this plasmid backbone and incorporated into the backbone with the aid of IS*CR1* and IS*26* elements.

**FIG 2:**
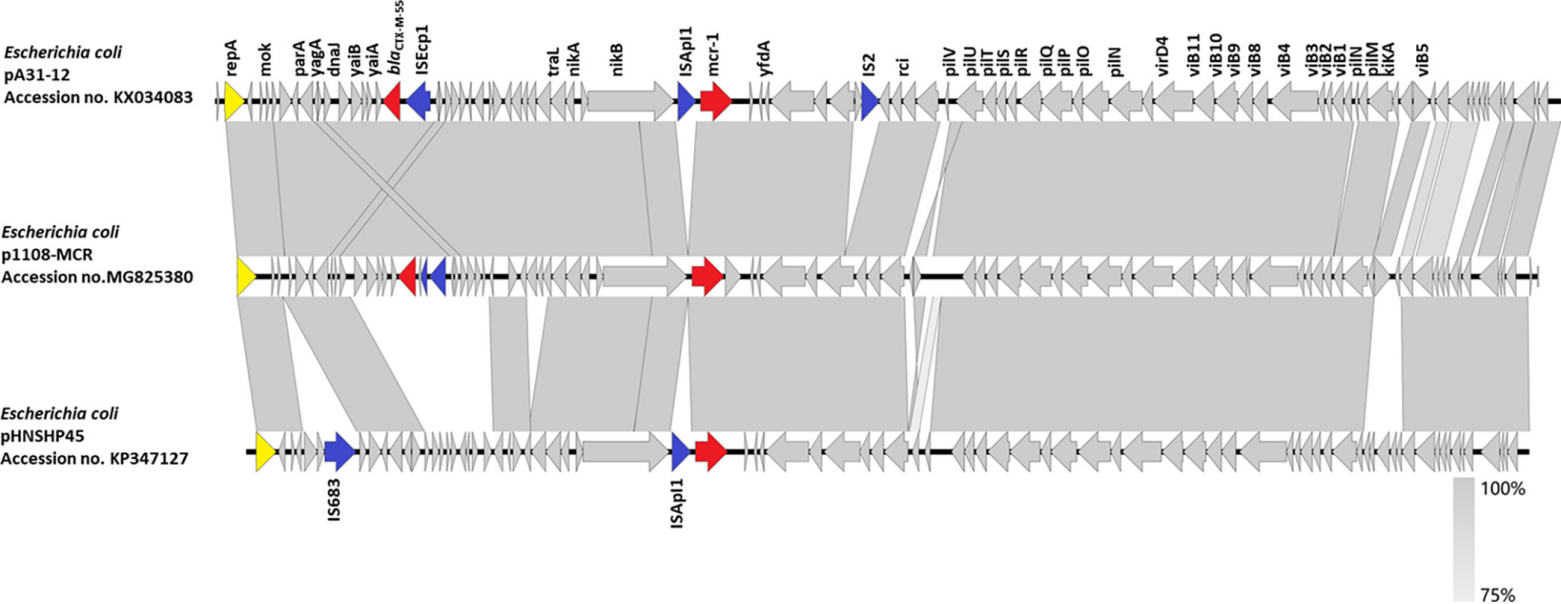
Schematic representation of the structure of the *mcr-1*- and *bla*_CTX-M-64_-surrounding sequences in plasmid p1108-MCR (MG825380). Results of sequence alignments with *mcr-1*- and *bla*_CTX-Ms_-associated genetic structures identified in *Eshcerichia coli* plasmid pA31-12 (KX034083) and E. coli plasmid pHNSHP45 (KP347127) are shown. CDSs without labels represent hypothetical proteins. The shaded parallelograms denote genetic regions that exhibit sequence homology among different segments. Light shading denotes regions with a lower level of sequence identity. Arrows indicate CDSs, with arrowheads indicating the direction of transcription: red, antibiotic resistance-encoding genes; blue, mobile elements; yellow, replication protein; gray, maintenance/stability functioning genes, or hypothetical proteins.

Resembling other IncI2-type plasmid backbones, p1108-MCR contains the gene encoding the RepA replicon protein, genes involved in plasmid stability (*yafA*, *mok*, *hok* and *yafB*), the chromosome (plasmid) partitioning protein (*parA* and *parB*), genes encoding proteins related to the type IV secretion complex (*virB* and *virD4*), as well as a plasmid-borne site-specific recombinase (*Rci*) gene and the conserved genes in all IncI2 plasmids, which have the pilus-encoding gene (*pil*) and the ones responsible for plasmid transfer (*tra*) (23). BLASTN comparison revealed that p1108-MCR had a structure highly similar to pSCS23 (KU934209), which was isolated from Salmonella enterica of chicken origin in China, and BA76-MCR-1 (KX013540), which was isolated in E. coli BA76 in a sacral wound swab in a patient in the Arabian Peninsula. In p1108-MCR, two acquired resistance genetic regions were identified. The *bla*_CTX-M-64_ gene was located in a 1,783-bp IS*Ecp1* transposition unit containing a truncated IS*Ecp1* (336 bp), *bla*_CTX-M-64_, orf*477*, and a 194-bp fragment. A 5-bp direct repeats (TTTTC) flanking the ΔIS*Ecp1* was presented in the IncI2 backbone. Moreover, the left inverted repeats (5′-CCTAGATTCTACGT-3′) and right inverted repeats (5′-CCTAAATTCCACGT-3′) were observed flanking ΔIS*Ecp1*, and a perfect IRR (5′-ACGTAGAATCTAGG-3′) was located upstream of orf*477*, which was similar to the configuration of pA31-12 (KX034083) (23). This configuration was widely presented in other IncI2 plasmids, such as pCTXM64_C0967 harboring *bla*_CTX-M-64_ and pHN1122-1 carrying the *bla*_CTX-M-55_ gene, indicating that these IncI2 plasmids originated from a common ancestor (24). However, the IS*683* and IS*Apl1* elements, which were commonly observed in other reported IncI2 types of plasmid harboring the *mcr-1* gene, were not found in plasmid p1108-MCR compared with pHNSHP45 (KP347127) (Fig. 2).

The backbone of p1108-IncY showed a high nucleotide sequence identity with the bacterial phage P1 and the phage P1-like region of pKP1226 (Fig. S2), suggesting that p1108-IncY was most likely to be lysogenized into a plasmid via phage sequences. Plasmid p1108-IncFIB harbored a transfer region, including *tra* and *trb* genes and also a virulence region from *iroBCDEN* of the salmochelin siderophore system, to *iucABCD* and *iutA* of the aerobactin iron transport system with a size of 62.1 kb, which was similar to that of pAPEC-O1-ColBM, p1ColV5155, and an IncF-type plasmid (pC59-153) (Fig. S3). Plasmid p1108-ermB revealed sequence similarities with pS68 and plasmid II, which were obtained from China and the United Kingdom, respectively. Moreover, two resistance genes (*emrB* and *aadA22*) and a class I integron (In155; AM261837) were found in p1108-ermB (Fig. S4). Plasmid p1108-CMY2 harbored the IS*Ecp1*-*bla*_CMY-2_ transposition unit, and no other resistance genes were present in this plasmid other than *bla*_CMY-2_ (Fig. S5). The last plasmid in this strain, p1108-Col, was a small ColpVC plasmid with a size of 2,096 bp, and no antimicrobial resistance gene or IS element was found in it (Table 1).

### Conclusion.

In conclusion, this study described the complete genetic features of seven plasmids in a foodborne CPE E. coli strain harboring 16 resistance genes, a phage P1-like region, and multiple virulence-related genes. Importantly, the concurrence of *bla*_NDM-1_ and *mcr-1* genes (in different plasmids) in retail chicken meat sample provides a warning that colistin- and carbapenem-resistant genes have been disseminated into food products. Considering the fact that colistin is the last choice for treating human and animal infections caused by MDR *Enterobacterales*, infections due to strains that simultaneously carry the *mcr-1* and CPE genes are expected to become almost untreatable. Effective surveillance and intervention approaches to control the transmission of such MDR plasmids are urgently required.

## MATERIALS AND METHODS

### Bacterial isolation.

E. coli isolate 1108 was obtained from chicken meat purchased from a supermarket in Shenzhen, Guangdong Province, China on 27 March 2017. This isolate was among a number of meropenem-resistant E. coli strains isolated from chicken samples using the following approach: 25 g of chicken sample were placed in a sterile homogeneous bag containing 50 mL of sterilized saline; 1 mL of homogenate was transferred to lactose broth and incubated at 42°C for 12 to 16 h; and 1 mL each of pre-enriched broth was transferred to a MacConkey agar plate supplemented with 0.5 μg/mL meropenem. Following incubation at 37°C for 16 h, two or three putative E. coli isolates were purified on MacConkey agar plates containing 0.5 μg/mL meropenem. The MALDI-TOF MS was used to identify E. coli isolate 1108 by Bruker MicroFlex LT mass spectrometer (Bruker Daltonics); the species identity of this strain was further confirmed by an API20E test strip (bioMérieux, Inc.).

### Antimicrobial susceptibility testing.

Antimicrobial susceptibility of strain 1108 was tested based on previous reports (25), following the guidelines of the Clinical and Laboratory Standards Institute (CLSI) (26). Twelve antibiotics (including colistin, meropenem, ceftazidime-avibactam, fosfomycin, kanamycin, chloramphenicol, nalidixic acid, amikacin, ciprofloxacin, cefotaxime, ampicillin, and trimethoprim-sulfamethoxazole) were tested. E. coli strain ATCC 25922 and Staphylococcus aureus ATCC 29213 were used as the quality control strain.

### Genetic characterization of E. coli strain 1108.

E. coli strain 1108 was subjected to screening for the presence of *mcr-1* genes and β-lactamase genes, including *bla*_NDM-1_ gene by PCR; primers were used as previously described (2, 27, 28). The genetic identity was confirmed by Sanger sequencing of purified PCR products (28106, Qiagen).

### Conjugation, S1-PFGE, and Southern hybridization.

A filter-mating experiment was carried out to test the transferability of resistance phenotypes of strain 1108. Overnight cultures of donor (E. coli 1108) and recipient (sodium-azide-resistant E. coli J53) were mixed together in a ratio of 4:1 and plated on a filter membrane (0.45 μm) on LB agar medium without selection. E. coli strain 1108 was expected to undergo conjugative transfer of two types of plasmid. For plasmid carrying the *bla*_NDM-1_ gene, MacConkey Agar containing meropenem (1 μg/mL) and sodium azide (200 μg/mL) was used for selection of transconjugants that have acquired such plasmid, followed by verification of the presence of the *bla*_NDM-1_ gene by PCR. For the *mcr-1* gene, Eosin Methylene Blue Agar containing colistin (2 μg/mL) and sodium azide (100 μg/mL) was used, followed by verification of the presence of *mcr-1* in the plasmid by PCR. The plasmid profiles were characterized by S1-nuclease PFGE using the Chef-Mapper system (Bio-Rad, USA). The locations of *bla*_NDM_ and *mcr-1* in E. coli strain 1108 and the corresponding transconjugants were identified by Southern hybridization, using digoxigenin-labeled probes in accordance with the manufacturer’s instructions. The whole genome of E. coli 1108 was sequenced and was then subjected to do multilocus sequence typing (MLST) according to the protocol at an online database (http://bigsdb.pasteur.fr/) for E. coli.

### Sequencing and bioinformatics analyses of plasmids.

To determine the complete nucleotide sequences of plasmids in E. coli strain 1108, the plasmids were extracted by the Qiagen Plasmid Midi kit (Qiagen, Germany) and were decoded by whole-plasmid sequencing using the Illumina Nextseq 500 and MinION platforms (Oxford Nanopore Technologies) as described previously (29). Briefly, paired-end Illumina reads (2 × 150 bp) and MinION long reads were generated with the NEBNext Ultra DNA Library Prep kit and Rapid Barcoding Sequencing kit, respectively. Hybrid assembly strategy was used to perform *de novo* assembly with Unicycler (30) combining short- and long-read data. Gene prediction and annotation were conducted by RAST (31) and edited manually. Alignment with complete sequences of plasmids available in the NCBI database was conducted with the BRIG (32) and Easyfig (33) tools.

### Data availability.

The completed plasmid sequences for p1108-NDM, p1108-MCR, p1108-emrB, p1108-CMY2, p1108-IncY, p1108-IncFIB, and p1108-Col were deposited in NCBI with accession numbers MG825381, MG825380, MG825377, MG825376, MG825379, MG825378, and MH425140, respectively.
