# Supplementary material for: Complete Genetic Analysis of Plasmids Carrying Multiple Resistance, Virulence, and Phage-Like Genes in Foodborne Escherichia coli Isolate
Source: Microbiol Spectr. 2023 Mar 21;11(2):e02820-22. doi: 10.1128/spectrum.02820-22 (PMC10101111; doi:10.1128/spectrum.02820-22)
Supplement: Supplemental file 1 — Supplemental material. Download spectrum.02820-22-s0001.pdf, PDF file, 0.9 MB [file spectrum.02820-22-s0001.pdf]

Supplementary Data

Complete genetic analysis of plasmids carrying multiple resistance, virulence and phage-like genes in foodborne *Escherichia coli* isolate

Xiaobo Liu[1], Ruichao Li[2], Edward Wai-Chi Chan[3], Sheng Chen[4]*

Running title: Complete genetic analysis of MDR plasmids

[1]National Engineering Laboratory for Deep Process of Rice and By-products, College of Food Science and Engineering, Central South University of Forestry and Technology, Changsha, 410004 Hunan China;
[2]Institute of Comparative Medicine, College of Veterinary Medicine, Yangzhou University, Yangzhou, Jiangsu Province, P. R. China;
[3]The State Key Lab of Chemical Biology and Drug Discovery, Department of Applied Biology and Chemical Technology, The Hong Kong Polytechnic University, Hung Hom, Kowloon, Hong Kong SAR;
[4]Department of Infectious Diseases and Public Health, Jockey Club College of Veterinary Medicine and Life Sciences, City University of Hong Kong, Kowloon, Hong Kong SAR.

*Corresponding author:
Sheng CHEN, Tel: 852-34425782, Email: shechen@cityu.edu.hk

Keywords: *bla*$_{NDM}$, *mcr-1*, Foodborne *E. coli*, Plasmid, Genetic analysis

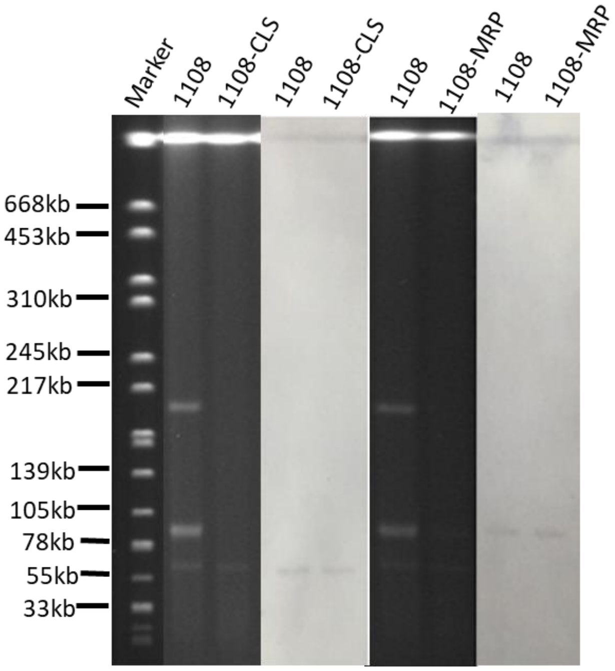

Figure S1. S1-PFGE and Southern hybridization of *E. coli* strains 1108 and the corresponding transconjugants. This has been reported in our previous study(1).

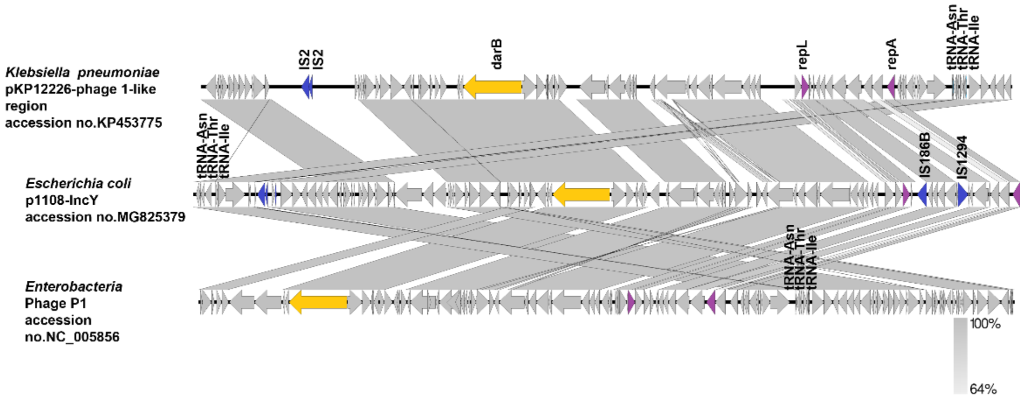

Figure S2. Sequence alignment of phage P1-like region of pKP1226 (KP453775), phage P1 (NC_005856) and plasmid p1108-IncY (MG825379). Bacteriophage P1 was from *Enterobacteria* and plasmid pKP12226 was carried by a *Klebsiella pneumoniae* strain K01-12226 recovering from a patient with bacteremia in South Korea(2). Light gray shading denotes shared regions of homology. Gray shading indicates homologies between the corresponding genetic loci in each plasmid. Arrows indicate CDSs, with arrowheads indicating the direction of transcription: purple, replicase genes; blue, mobile elements; yellow, DNA methylase gene *darB*, gray, plasmid scaffold regions that are common among the plasmids.

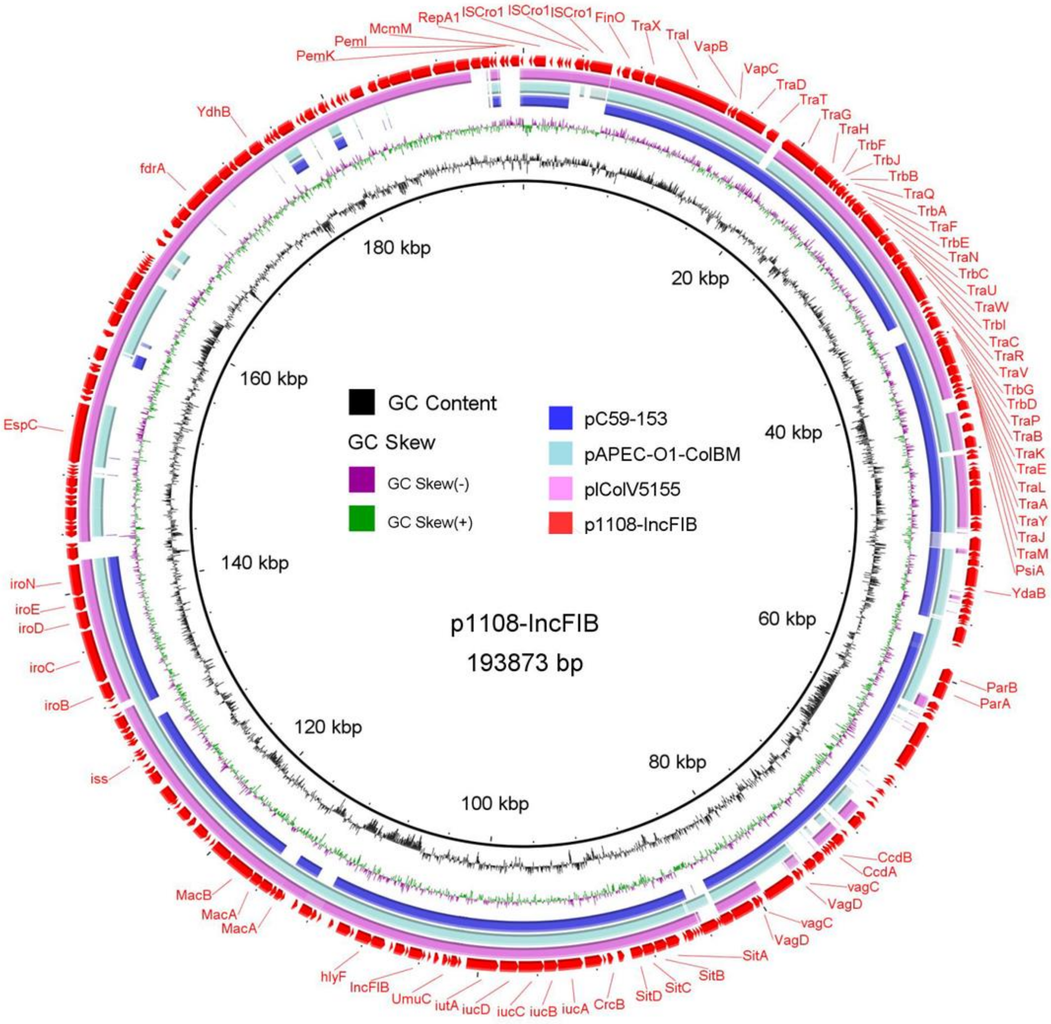

Figure S3. Sequence alignment of pC59-153 (KJ484636), pAPEC-O1-ColBM (DQ381420), p1ColV5155 (CP005931) and p1108-IncFIB (MG825378). Plasmid pC59-153 was carried by *E. coli* C-59 strain isolating from a chicken fecal sample(3), p1ColV5155 was carried by avian pathogenic *E. coli* (APEC) strain IMT5155 which was recovered from a chicken with avian colibacillosis in Germany(4) and pAPEC-O1-ColBM was harbored by APEC strain O1 (O1:K1) isolating from a turkey with colibacillosis(5). p1108-InFIB was used as a reference. The outer circle with red arrows denotes annotation of the reference sequence, the gaps represent sequences that exist only in the reference plasmid.

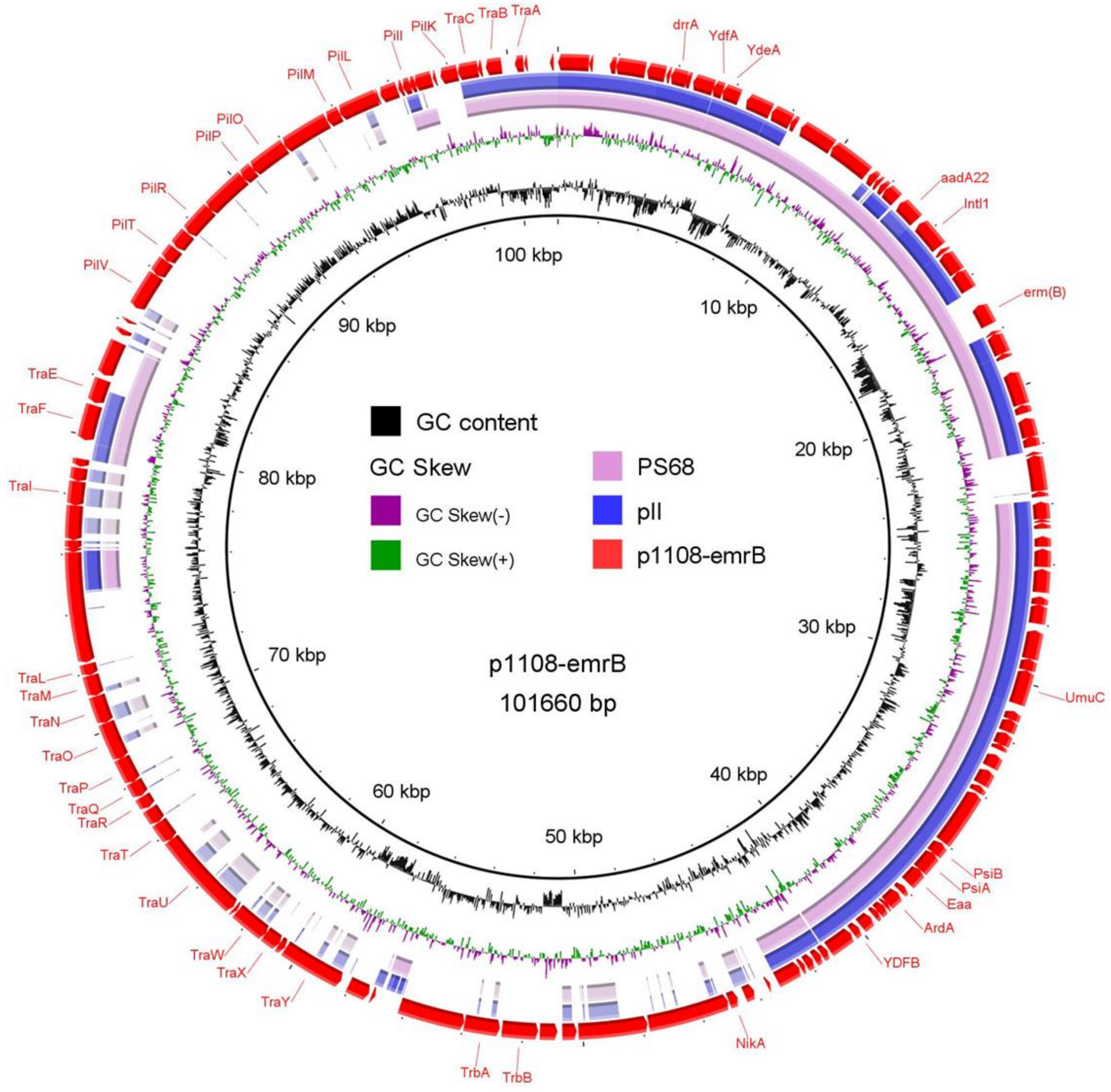

Figure S4. Sequence alignment of pS68 (KU130396), pII (LT795504) and p1108-emrB (MG825377). Plasmid pS68 was harbored by an *E. coli* isolating in China and plasmid II was isolated from an *E. coli* strain KV7 obtaining from pig feces in United Kingdom. p1108-emrB was used as a reference. pS68 and pII respectively exhibits 50% and 43% sequence similarity with the reference. The outer circle with red arrows denotes annotation of the reference sequence, the gaps represent sequences that are missing when compared to the reference plasmid.

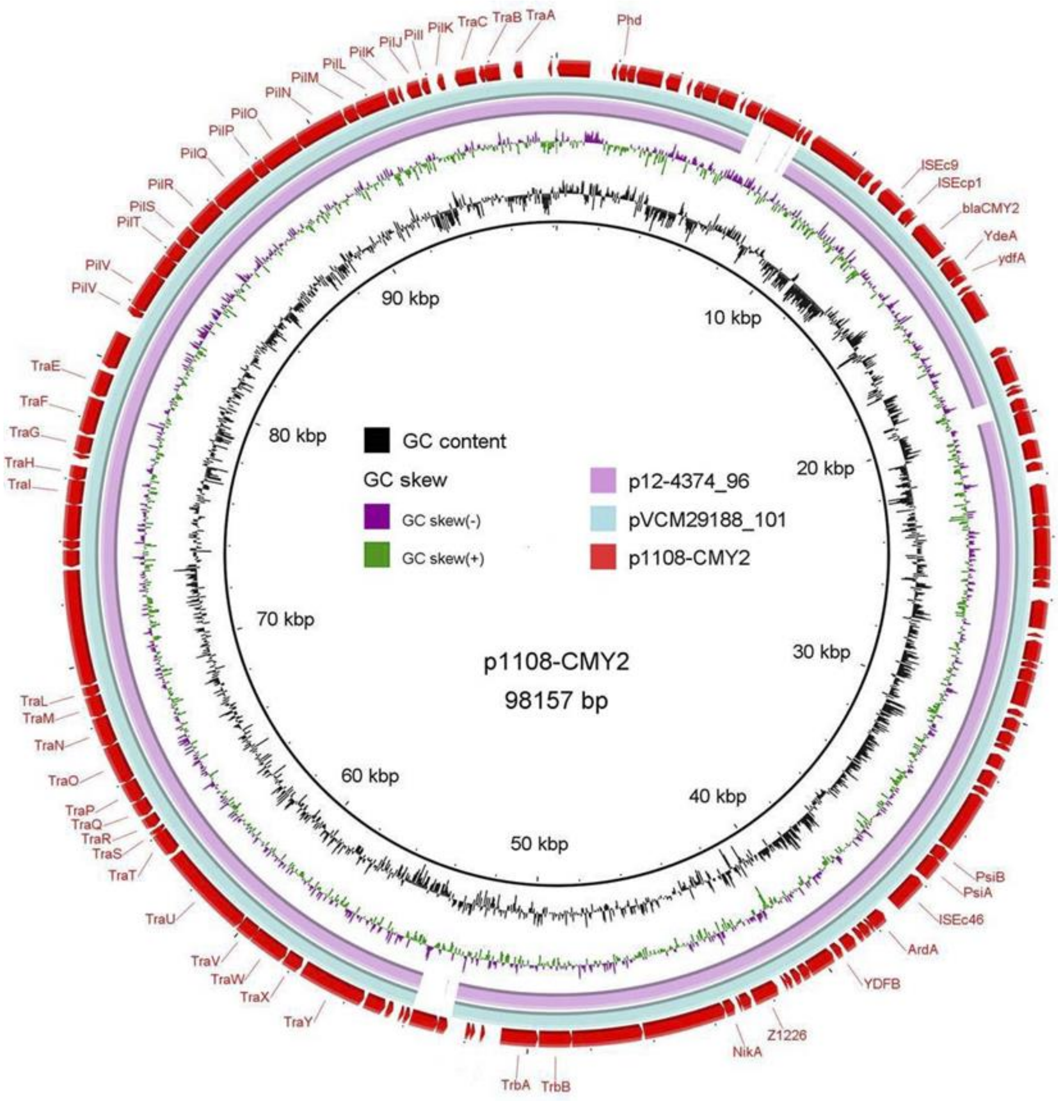

Figure S5. Sequence alignment of p12-4374_96(CP012929), pCVM29188_101(CP001121) and p1108-CMY2(MG825376). The latter one was used as a reference. Plasmid p12-4374_96 was carried by a *Salmonella* strain 12-4374 in Canada isolating from the human stool(6) and pCVM29188_101 was harbored by a *Salmonella* strain CVM29188 obtaining from poultry(7). The outer circle with red arrows denotes annotation of the reference sequence, the gaps represent sequences that were found to be missing when compared to the reference plasmid.

References:

1. Liu X, Geng S, Chan EW, Chen S. 2019. Increased prevalence of *Escherichia coli* strains from food carrying *bla*$_{NDM}$ and *mcr-1*-bearing plasmids that structurally resemble those of clinical strains, China, 2015 to 2017. Eurosurveillance 24.http://dx.doi.org/10.2807/1560-7917.ES.2019.24.13.1800113
2. Shin J, Ko KS. 2015. A plasmid bearing the *bla*$_{(CTX\text{-}M\text{-}15)}$ gene and phage P1-like sequences from a sequence type 11 *Klebsiella pneumoniae* isolate. Antimicrobial Agents and Chemotherapy 59:6608-10.http://dx.doi.org/10.1128/AAC.00265-15
3. Wang J, Stephan R, Power K, Yan Q, Hachler H, Fanning S. 2014. Nucleotide sequences of 16 transmissible plasmids identified in nine multidrug-resistant *Escherichia coli* isolates expressing an ESBL phenotype isolated from food-producing animals and healthy humans. Journal of Antimicrobial Chemotherapy 69:2658-68.http://dx.doi.org/10.1093/jac/dku206
4. Zhu Ge X, Jiang J, Pan Z, Hu L, Wang S, Wang H, Leung FC, Dai J, Fan H. 2014. Comparative genomic analysis shows that avian pathogenic *Escherichia coli* isolate IMT5155 (O2:K1:H5; ST complex 95, ST140) shares close relationship with ST95 APEC O1:K1 and human ExPEC O18:K1 strains. PLoS One 9:e112048.http://dx.doi.org/10.1371/journal.pone.0112048
5. Johnson TJ, Johnson SJ, Nolan LK. 2006. Complete DNA sequence of a ColBM plasmid from avian pathogenic *Escherichia coli* suggests that it evolved from closely related ColV virulence plasmids. Journal of Bacteriology 188:5975-83.http://dx.doi.org/10.1128/JB.00204-06
6. Labbe G, Edirmanasinghe R, Ziebell K, Nash JH, Bekal S, Parmley EJ, Mulvey MR, Johnson RP. 2016. Complete Genome and Plasmid Sequences of Three Canadian Isolates of Salmonella enterica subsp. enterica Serovar Heidelberg from Human and Food Sources. Genome Announc 4.http://dx.doi.org/10.1128/genomeA.01526-15
7. Fricke WF, McDermott PF, Mammel MK, Zhao S, Johnson TJ, Rasko DA, Fedorka-Cray PJ, Pedroso A, Whichard JM, Leclerc JE, White DG, Cebula TA, Ravel J. 2009. Antimicrobial resistance-conferring plasmids with similarity to virulence plasmids from avian pathogenic Escherichia coli strains in Salmonella enterica serovar Kentucky isolates from poultry. Appl Environ Microbiol 75:5963-71.http://dx.doi.org/10.1128/AEM.00786-09
